# Suction Drain Volume following Axillary Lymph Node Dissection for Melanoma—When to Remove Drains? A Retrospective Cohort Study

**DOI:** 10.3390/jpm12111862

**Published:** 2022-11-07

**Authors:** Raimund Winter, Alexandru Tuca, Paul Wurzer, Caroline Schaunig, Isabelle Sawetz, Judith C. J. Holzer-Geissler, Daniel Georg Gmainer, Hanna Luze, Herwig Friedl, Erika Richtig, Lars-Peter Kamolz, David Benjamin Lumenta

**Affiliations:** 1 Research Unit for Tissue Regeneration, Repair and Reconstruction, Division of Plastic, Aesthetic and Reconstructive Surgery, Department of Surgery, Medical University of Graz, Auenbruggerplatz 5, A-8036 Graz, Austria; 2 Institute of Statistics, Graz University of Technology, Kopernikusgasse 24, A-8010 Graz, Austria; 3 Department of Dermatology, Medical University of Graz, Auenbruggerplatz 8, A-8036 Graz, Austria; 4 COREMED—Cooperative Centre for Regenerative Medicine, Joanneum Research GmbH, Neue Stiftingtalstraße 2, A-8010 Graz, Austria

**Keywords:** skin neoplasms, melanoma, lymph node excision, drainage, seroma therapy

## Abstract

Postoperative complications such as seroma formation and wound-site infection occur following completion axillary lymph node dissection (ALND) for melanoma. We analyzed the impact of time-to-drain removal and drainage volume on seroma formation after ALND. We retrospectively analyzed data from 118 patients after completion ALND for melanoma. Primary endpoints were daily amount of drainage volume, seroma formation and time-to-drain removal. Secondary endpoints included patient-related, disease-specific and perioperative parameters as well as the number of histologically analyzed lymph nodes and surgical complications graded by the Clavien–Dindo classification (CDCL). Statistical analyses were performed using logistic regression models. Drain removal around the 8th postoperative day was statistically associated with a lower risk for the occurrence of seroma formation (*p* < 0.001). Patients with an increased drainage volume during the early postoperative days were more prone to develop seroma after drain removal. With 49% (CDCL I and II), most complications were managed conservatively, while only 5.9% (CDCL III) required revision surgery (CDCL overall: 55.9%). ALND is a safe procedure with a low rate of severe CDCL III type of complications. To decrease seroma evacuation, our results imply that drains should be removed around the 8th postoperative day to reduce the risk of infection, readmission or prolonged hospitalization.

## 1. Introduction

With the change of guidelines, positive axillary sentinel node detection is no longer the sole trigger for completion axillary lymph node dissection (ALND) in melanoma. Current evidence considers the detection of a clinically or imaging-confirmed pathological lymph node important before performing ALND [1,2,3,4]. With an unclear benefit of ALND for overall survival, ALND positively impacts relapse-free survival [5], but is often associated with complications such as seroma formation, wound infections or lymphedema [6].

Seroma formation is a common complication after ALND for melanoma [7,8,9], requiring percutaneous evacuations [10] during outpatient visits; this is also associated with a decreased quality of life. Routinely applied suction drains can reduce the evacuation of seroma formation by approximately 50% [11], while the risk for developing a seroma remains omnipresent. Most centers apply clinical protocols for removal of drains based on a set postoperative timepoint or defined (daily) drainage volume [12]. On the one hand, premature removal increases the risk for seroma formation; on the other hand, late removal increases the chances of developing wound infections. At the time of submission, we were unaware of the existence of evidence-based guidelines describing adequate time or volume limits for defining an optimal time-point for removal of intraoperatively placed drains after ALND for melanoma [12,13].

While seroma formations after ALND in breast cancer are well-described, the aim of this study was to detect the impact of time-to-drain removal on the amount of aspirated seroma fluid after ALND for melanoma. We wanted to retrospectively analyze our patients’ data and to provide recommendations for prevention of seroma formation based on our data.

## 2. Materials and Methods

We retrospectively collected data from 118 patients receiving completion ALND for melanoma at the division of plastic, aesthetic and reconstructive surgery at the university hospital in Graz between January 2005 and October 2017. Included patients had a histologically confirmed melanoma and a histologically positive sentinel node, underwent ALND (level I–III) and had daily drainage volume records over a two-week period. Prior to data collection, we obtained ethical approval (institutional review board # 29-607 ex 16/17). Data collection and analysis were performed in accordance with the rules set by the Declaration of Helsinki. This work has been reported in line with the STROCSS criteria [14].

Primary endpoints were daily/total drainage volume during hospitalization as well as the drainage volume 24 h prior to drain removal, time-to-drain removal, and evacuation of seroma after drain removal. Seroma was defined as any accumulation of fluid in the axilla that required evacuation.

Secondary endpoints included demographic data of all patients (age, sex, body mass index (BMI), smoking, diabetes mellitus, allergies, Breslow Index), intraoperative parameters (surgical procedure time, site), amount of histologically analyzed lymph nodes and surgical complications (graded by the Clavien–Dindo classification, CDCL [15,16]).

In all operative sites of ALND, a negative-pressure suction drain system was placed (Exulock large 400^®^, Fresenius Kabi AG, Bad Homburg, Germany), and all vacuum flasks were exchanged daily to maintain the negative pressure, which also served as a record for the daily amounts of drainage fluid (in mL). The time-to-drain removal was documented in postoperative days (d). Removal of drains was performed if the amount of drained fluid was <50 mL per 24 h or on the 14th postoperative day, whichever came first. Patients were discharged 24 h after drain removal and were followed-up as outpatients. During outpatient visits, evacuation of seroma was defined as any clinically palpable fluid accumulation at the operative site for ALND, and we recorded the aspirated seroma volume from percutaneous aspirations.

Statistical analyses were done by a professional statistician and were based on a linear logistic regression model that was trained by demographic data and “and other unique/one-time aspects” (e.g., age, gender, body mass index, Breslow index, operative time) of the 118 patients. In addition to this model, an additive logistic model [17] was applied to study the impact of the daily monitored redon discharge and also to allow for a smooth effect of time-to-drainage removal.

A *p* < 0.01 was considered statistically significant. Demographic data were described as median (interquartile range, iqr).

## 3. Results

### 3.1. Patient Characteristics

The median (interquartile range) age was 62.3 (23.6) years, and 58% were male. A total of 13 patients were smokers (11%), 11 had diabetes mellitus type 2 (9%), 20 had known allergies (17%), and the median BMI was 27.1 (5.6). The median Breslow Index was 2.3 (2.6) mm, the median surgical procedure time was 114.5 (57.5) min, the median number of histologically affected (positive) lymph nodes was 2 (2), and the median number of resected axillary lymph nodes was 15 (9). There was no significant impact of the Breslow Index, number of affected lymph nodes, surgical procedure time, age, sex and BMI on the evacuation of seroma after drain removal. The median duration of the drainage was 10 (6.7) days.

### 3.2. Complications and Revision Surgery

A total of 66 patients out of 118 (55.9%) had complications according to the CDCL (Table 1), of which the most common complications were seroma formation 38 (32.2%), impaired wound healing 16 (13.5%), and wound infection 12 (10.2%). The highest share of the CDCL was complications recorded as grade I and II, with 46 (69.7%) and 12 (18.2%), respectively. Only 5.9% of all patients needed revision surgery due to vast hematoma formation, infection or the formation of high-volume seroma over the course of several weeks.

**Figure 1:**
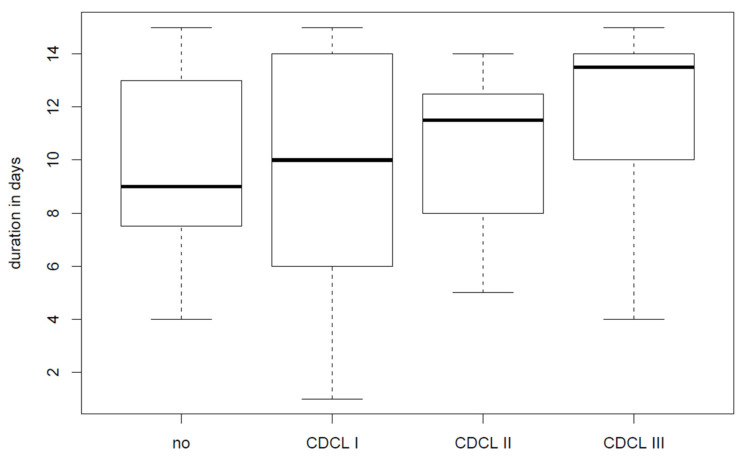
The box plots show the relationship of the median (bold black horizontal lines in box, which represents the interquartile range) drain indwelling time (=time-to-drain removal) and the increasing degrees of complications classified by the Clavien–Dindo Classification (CDCL). Patients with no complications had their drains removed at day 9 (5.5), CDCL I patients at day 10 (8), CDCL II patients at day 12 (3.75) and CDCL III patients at day 14 (3). Data are depicted as median (interquartile range).

All patients with wound infections had a drain indwelling time of at least 7 days (time-to-drain removal ≥ 7 days). The longer the drain indwelling time, the higher the CDCL grade of complications was, as shown in Figure 1.

**Figure 2:**
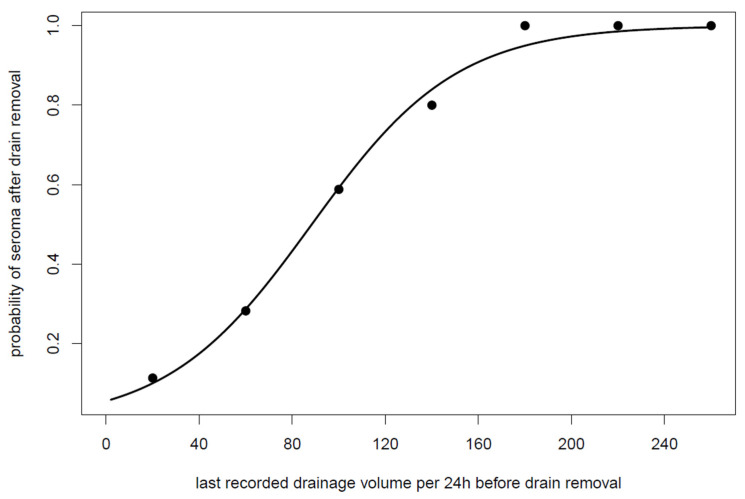
The probability of seroma puncture depending on the drainage flow rates in the last 24 h before removal of the drainage with empirical proportions observed in drainage volume intervals of 40 mL (shown as big dots). The logistic regression shows that a high drainage volume before removal has a high probability of developing seroma needing evacuation in the future.

Of all patients, 38 (32.2%) developed seroma after drain removal. The last recorded drainage volume (mL output of the last 24 h before drain removal) was a statistically relevant and significant predictor for the evacuation of seroma (*p* < 0.0001) as shown in Figure 2.

**Figure 3:**
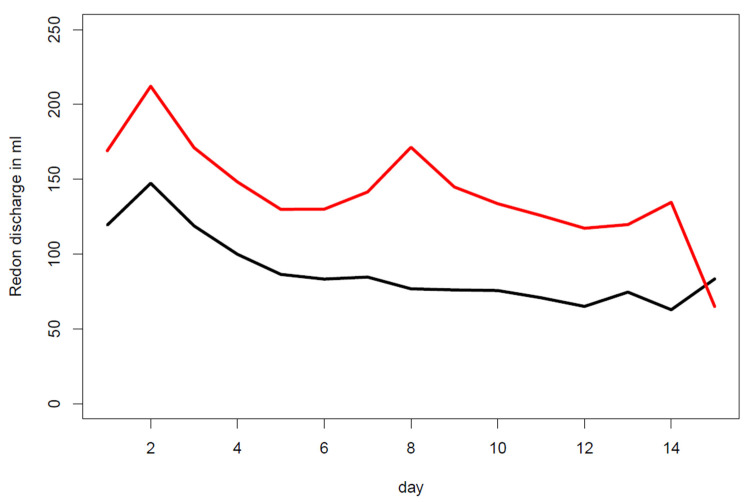
Average daily drainage volumes over time of patients with seroma evacuation (red curve) vs. patients not developing a seroma after drain removal (black curve). Both curves show an increase at day 2 post surgery. The drainage volume of patients who did not develop seroma declined steadily. Patients who developed a seroma showed an increase in drainage volume around day 8.

Patients who required a percutaneous evacuation of a seroma in the outpatient clinic had an average of 60 mL or more drainage output at the day of drain removal than patients who developed no seroma. The pattern of the average daily drainage volumes over time was similar in both groups as shown in Figure 3.

### 3.3. Drain Indwelling Time

**Figure 4:**
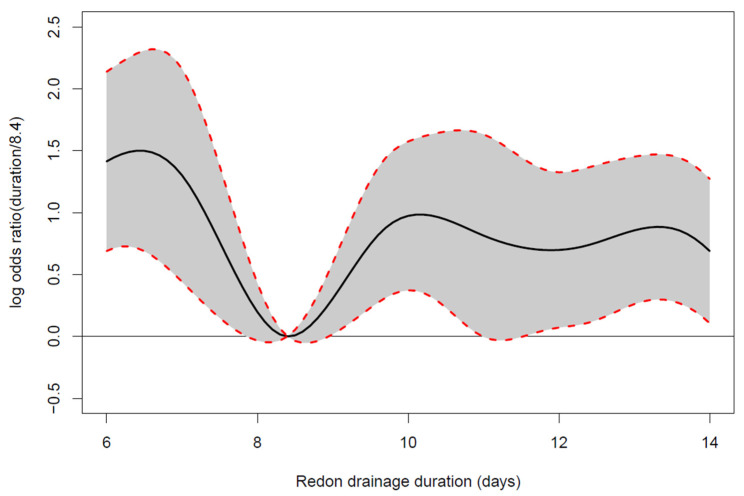
Estimated log odds ratios comparing the probability for further seroma evacuation at any other day-to-drain removal (drain indwelling time) vs. the time of drain removal at 8.4 days with pointwise 95% confidence intervals. The odds ratio of 0 implicates the lowest possible probability of developing a seroma. Removal before and after day 8.4 was associated with higher log odds ratios for seroma evacuation (black solid line).

Our primary endpoints—the drain indwelling time (*p* = 0.003), the daily drainage volume (*p* < 0.0001) and the last recorded daily drainage volume before drain removal (*p* = 0.001)—were significant predictors for the future need of evacuation of seroma in an additive logistic regression model [17]. The statistical model used confirmed that the drain removal between the 8th and 9th postsurgical day (more precisely on day 8.4) resulted in the lowest probability for the requirement of seroma evacuation (Figure 4).

Removal before and after day 8.4 was associated with higher log odds ratios for seroma evacuation (black solid line Figure 4). Only within a very close timely vicinity of 8.4 days, the 95% confidence intervals slightly overlapped the neutral line below zero; this indicated that all other drain indwelling times were significantly worse than the one of 8.4 days, resulting in higher odds for the need of seroma evacuation after drain removal. Of note was also that log odds ratios beyond 10 days of drain indwelling time were relatively constant.

## 4. Discussion

Our single-center analysis indicated that drain removal around the 8th postoperative day significantly reduced the statistical risk for requiring seroma evacuation after drain removal after ALND for melanoma. Patients with high drainage volumes—as measured by daily drain outputs—were more likely to develop a seroma after drain removal. An indicator in this context was the last 24 h drainage output before drain removal—albeit an obvious, however unpublished, clinical parameter as far as we know. In our collective, we found none of the “usual suspects” to influence postoperative complications: neither patient factors such as BMI and age nor disease/surgical factors such as Breslow Index, number of affected lymph nodes and operative time had a significant impact on evacuation of seroma. While “major” complications (CDCL ≥ III) were rare, ALND was associated with a high risk for developing “minor” complications (CDCL I-II) and one-tenth of all analyzed patients developed wound-site related problems (local infection/inflammation).

The overall complication rate after ALND for melanoma is reported to range between 11% [18] and 53% [19]. Only 5.9% of all our cases required operative revisions under general anesthesia. Most postoperative records in our sample were CDCL I (69.7%); this subset of postoperative complications is often not recorded in the body of previous literature sources on ALND after melanoma. Furthermore, the requirement for operative revision was not addressed in a previous study with a comparable complication rate of 53% [19]: the cited publication noted 21% postoperative infections, 15% seromas requiring evacuation and 4% skin-tip necroses, as well as 6% wound-healing disorders [19]. In our opinion, reoperations are a decisive factor for evaluation of patient safety and should be recorded for analysis of postoperative complications of ALND in melanoma [20]. Based on our data, we can consider ALND to be a safe procedure among patients with melanoma, with an overall complications rate of 55.9%, of which most are “mild” (CDCL I—69.7%) and “moderate” (CDCL II—18.2%), and there is only a low rate of “severe” (CDCL III—12.1%) complications.

The published complication rates after ALND for melanoma suggest that the lack of a uniform classification scheme and lack of published reoperation rates were the main reasons for their variability and range (11–53%). Since the CDCL allows recording of complications in detail, we highly suggest this classification should be used for further evaluations to allow for a better comparison of postoperative complications.

Seromas requiring repetitive evacuations can be painful, can prolong hospital stays and should be avoided. In our patient cohort, a seroma was recorded in 32% of cases. The literature sources on postoperative seroma formation describe ranges between 2% [18] and 41% [21].

Based on our continuous daily documentation of drainage output, we were able to predict the development of an axillary seroma and to detect our finding of the 8th postoperative day to be a statistically optimal timepoint to remove the drains and subsequently reduce the evacuation of seroma formation. However, this finding applied to our collective, and might be different when a comparable evaluation takes place in a future prospective trial with different postoperative protocols (e.g., different drainage output or timely cut-off value to trigger drain removal).

Most literature regarding seroma formation after ALND can be found for breast cancer patients. In the literature, the use of drains after ALND and when to remove them is still under debate [11]. Most authors remove the drains early on the first day after surgery if the drainage volume is ≤50 mL per 24 h or the day after without regard to the drainage volume [22,23]. If the drain is removed on a later day, the drainage volume is not considered as a factor regarding the removal [24,25]. Additionally, all of our melanoma patients had a positive sentinel node, meaning they already had one axillary surgery, whereas with breast cancer, most often only a needle biopsy of a pathological lymph node is performed prior to ALND. Furthermore, the objective observation of the drainage volume under inpatient conditions for 14 days is generally hardly described in the literature. Our data are therefore generally very rare, and a direct comparison of our collective to breast-cancer patients in the literature cannot be made.

The benefit of a completion lymph node dissection (CLND) in patients with sentinel-node metastases is under constant debate. Recent data suggest limited application of CLND in patients with clinically confirmed lymph node metastasis. None of the presented studies could show differences for survival between CLND vs. “watch and wait” (clinical observation with no intervention) in patients with microscopic sentinel lymph node metastasis [4]. Melanoma-specific survival in patients with sentinel-node metastases was not significantly better for the patients receiving CLND vs. for the ones without CLND. Patients with CLND, however, benefitted from regional disease control and histologic prognostic information for therapeutic management [26]. It is essential to discuss potential benefits and risks of CLND with every single patient before making a joint decision for carrying out the treatment plan [2]. Due to the questionable value of CLND, it is even more important to reduce postoperative complications such as seroma formation.

The advantage of our study was the standardized recording of daily drainage output and postoperative complications, reducing the possible limitations inherent to single-center analyses with retrospective designs. Nevertheless, this advantage is also one limitation of our study. In particular, the defined cutoff of drain removal was a result of a historically based development, but not one of empirical decision-making; it was therefore a unique opportunity to review and consequently change such a practice after our audit.

## 5. Conclusions

ALND is a safe procedure with a low rate of CDCL III complications requiring revision. Based on our collective’s data, we were able to identify the 8th postoperative day to be statistically optimal for drain removal after ALND for melanoma to reduce the evacuation of seroma formation and consequently decrease the requirement for percutaneous seroma evacuations or prolonged length-of-hospital stays. Our hospital protocol for drain removal has been adapted accordingly.

## Figures and Tables

**Table 1:** Patients’ information regarding complications. Out of 118 patients, 66 (55.9%) developed a complication.

Complications (Clavien–Dindo Classification)	Count (Overall)	Percentage (Complication)	Percentage (Overall)
Complication	66 (118)	100%	55.9%
Grade I	46 (66)	69.7%	39%
Grade II	12 (66)	18.2%	10.2%
Grade IIIa	1 (66)	1.5%	0.8%
Grade IIIb	7 (66)	10.6%	5.9%
Grade IVa	0	0%	0%
Grade IVb	0	0%	0%
Grade V	0	0%	0%

## Data Availability

The data presented in this study are available on request from the corresponding author. The data are not publicly available due to patients’ data privacy.
